# Experiential learning and mentorship as the foundation of clinical epidemiology training during infectious diseases fellowship: Response to “Training infectious diseases fellows for a new era of hospital epidemiology”

**DOI:** 10.1017/ash.2022.38

**Published:** 2022-04-11

**Authors:** Shandra R. Day, Mohammad Mahdee Sobhanie, Nora E. Colburn, Christina Liscynesky

**Affiliations:** Division of Infectious Diseases, Department of Internal Medicine, The Ohio State University Wexner Medical Center, Columbus, Ohio

## Abstract

A specific, clinical-epidemiology, month-long rotation for all infectious disease fellows as well as a 1-year subspecialty track provides education in clinical epidemiology during infectious disease fellowship training. We describe the educational process created at our institution to provide this training.

Infectious diseases (ID) physicians may have a formal compensated role in hospital infection prevention programs. However, in the era of COVID-19, all ID physicians have been required to mitigate infection control issues. We created a specific, clinical-epidemiology (EPI), month-long rotation for all of our ID fellows as well as a 1-year subspecialty EPI track for fellows interested in a career as a Hospital Epidemiologist. The recent commentary by Martin and Snyder^1^ outlined an educational framework supported by the Accreditation Council for Graduate Medical Education (ACGME) and the Society for Healthcare Epidemiologists of America (SHEA). Here, we have outlined the educational process that began in 2019–2020 academic year at The Ohio State University Wexner Medical Center (OSUWMC). Education stems from excellent teaching and real-world experience. Our institution has a robust EPI department, consisting of an administrative director, 4 medical directors, seventeen infection preventionists (IPs) including 3 senior IPs, 5 high-level disinfection (HLD) analysts, a data manager, and a program coordinator. The medical directors have focused oversight of the various patient populations, and they work closely with the IPs assigned to those locations.

As an overview, OSUWMC is a large, academic, quaternary-care, integrated health system comprising 1,600 beds (including 210 ICU beds and 204 PCU beds). The system includes University Hospital (a level I trauma and burn center, liver and kidney transplantation programs, labor and delivery with an adjacent 49 bed level III neonatal intensive care unit managed by Nationwide Children’s Hospital), the James Cancer Hospital (the Comprehensive Cancer Center Hospital with a bone marrow transplant unit), Ross Heart Hospital (with cardiology, cardiac and vascular surgery, and heart and lung transplantation programs), Brain and Spine Hospital (for neurology and neurosurgery), Dodd Rehabilitation Hospital, Harding Hall (an inpatient psychiatric unit), Talbot Hall (an inpatient addiction treatment unit), East Hospital (a community-based hospital with orthopedic surgery), and ∼200 primary, specialty, and subspecialty clinics including 4 ambulatory surgery centers.

## Clinical epidemiology training during infectious diseases fellowship

### Epidemiology rotation

The EPI rotation is a required 1-month-long rotation during the second year of ID fellowship. Fellows attend weekly hospital acquired infection (HAI) surveillance meetings, and Infection Prevention Committee (IPC) meetings. They learn one-on-one with IPs, and they are required to present an article for monthly journal club and successfully complete a quality improvement project (Table 1). Completed projects include evaluation of *Clostridioides difficile* (CDI) testing, a clinical review of candidemia cases, and a clinical review of carbapenem-resistant *Enterobacterales* colonization in solid-organ transplant patients.

**Table 1.** Epidemiology Fellow Rotation and Track Curriculum

Specific Activity	Rotation	Track
**System meetings**		
Weekly surveillance	X	X
Monthly IPC meeting	X	X
Staff meeting		X
Task force meetings including CLABSI, CAUTI, and CDI	X	X
Waterborne pathogen workgroup		X
Emerging and infectious pathogens workgroup	X	X
**Individual meetings with IPs**		
Department overview and scope	X	X
Construction/Facilities	X	X
Water management and air quality	X	X
Surveillance and process measure prevention: CAUTI, CLABSI, VAP, and SSI	X	X
Unit rounds	X	X
HLD and sterilization	X	X
Ambulatory infection prevention	X	X
High-risk areas—BMT, HD, endoscopy, etc	X	X
Influenza and TB plan	X	X
Cluster & outbreak investigation/exposures	X	X
Micro reports and CDI/MDRO pathogens	X	X
**OSUWMC policy review**		
COVID-19 resources/policies	X	X
Hand hygiene policy	X	X
Bloodborne pathogen exposure control policy		X
Cleaning and low-level disinfection policy		X
Employee health services infection prevention policy		X
Infection prevention plan	X	X
Isolation policy		X
TB control plan		X
Ambulatory infection prevention plan		X
Aseptic technique policy		X
HLD		X
HD infection prevention policy		X
**Required reading**		
CDC COVID-19 infection control guidance for healthcare professionals^5^		X
CDI guidelines^6^	X	X
Compendium of strategies to prevent healthcare-associated infections in acute care hospitals^7^	X	X
Guidance for infection prevention and healthcare epidemiology programs: healthcare epidemiologist skills and competencies^8^	X	X
Guideline for hand hygiene in healthcare settings^9^	X	X
Duration of contact precautions for acute care settings^10^	X	X
SHEA expert guidance: Outbreak response and incident management^11^	X	X
Review NHSN definitions^12^		X
Guideline for isolation precautions: preventing transmission of infectious agents in healthcare settings^13^		X
**Required coursework**		
*Primer on Healthcare Epidemiology, Infection Control and Antimicrobial Stewardship: Online ID Fellows Course* ^4^	X	X
SHEA/CDC training certificate course in healthcare epidemiology		X
Present EPI journal club	X	X
**Academic project**		
Quality improvement project	X	X
Abstract/Manuscript submission		X

Note. IPC, infection prevention and control; CLABSI, central-line–associated bloodstream infection; CAUTI, catheter-associated urinary tract infection; CDI, *Clostridioides difficile* infection; VAP, ventilator-associated pneumonia; SSI, surgical-site infection; HLD, high-level disinfection; BMT, bone marrow transplantation; HD, hemodialysis; TB, tuberculosis; MDRO, multidrug-resistant organism; NHSN, National Health Safety Network; SHEA, Society for Healthcare Epidemiologists of America; CDC, Centers for Disease Control and Prevention; EPI, clinical epidemiology.

### Epidemiology track

The EPI track was developed for second-year fellows interested in pursuing a career in hospital epidemiology with the goal of exposing and teaching the fellow the knowledge and skills necessary to be both a successful leader and expert in infection prevention. This program not only entails learning about specific HAI definitions as determined by the CDC but also how to effectively interact with administrators, physicians, facilities personnel, clinical staff, and public health officials while building their expertise in systems-based practice. The fellow learns how to construct health system–wide protocols, to determine and respond to exposures of infectious agents, to investigate new products related to infection prevention. They learn about facility systems (ie, water and airflow management), and they implement quality improvement projects. Because OSUWMC is a quaternary-care, integrated health system, the fellow learns the different requirements needed to ensure a safe environment for these unique populations such as bone marrow and solid-organ transplant patients, patients with acute leukemia, dialysis, patients in congregate settings, etc.

The EPI fellow has the opportunity to work alongside the entire EPI team during any hospital outbreak that occurs during the year regardless of what rotation they are on (Table 1). The fellow attends meetings with hospital administration and key stakeholders related to the outbreak, performs background research. Each fellow is responsible for helping with the outbreak interventions and timeline construction as well as any academic abstracts and/or manuscripts. Thus far, 2 fellows have successfully completed the track, and both actively participated in major outbreak investigations.^2,3^ The COVID-19 pandemic provided the unique opportunity for the medical directors to include the fellow in senior leadership and command center meetings as well as smaller multidisciplinary workgroup discussions and decision making.

Finally, this yearlong track allows the development of a long-term mentoring relationship between the fellow and the medical directors. While one medical director takes the lead on organizing the track, the fellow is actively mentored by all 4 faculty. This arrangement allows the fellow to observe various leadership styles and to learn the importance of collaboration and teamwork in systems-based practice.

## Core competencies and activities

### Surveillance and reporting

The Clinical Epidemiology Department meets weekly for 2 hours to review HAI surveillance. Cases of possible ventilator associated pneumonia (PVAP), central-line–associated bloodstream infection (CLABSI), catheter-associated urinary tract infection (CAUTI), and surgical site infection (SSI) are presented to the medical directors. Opportunities for HAI reduction are identified and discussed. HAI data are shared as close to real time as possible, with a formal monthly report to leadership and at the IPC. The enterprise-wide IPC has been established, and 5 other committees focus on specialized populations. Rotation fellows participate in weekly surveillance meetings to understand how surveillance is conducted, including multidisciplinary discussions and internal and external reporting, and to understand the differences between clinical and surveillance definitions. Fellows also meet individually with the IPs to review NHSN definitions, HAI process measure prevention, and required reporting to state and local health departments. The fellow is expected to attend at least 1 meeting of the system-wide HAI reduction task force to obtain a deeper understanding of the data and reporting including external benchmarking using SIR data. They are also expected to discuss strategies for HAI reduction. In addition, the EPI track fellow attends weekly surveillance meetings when they are not assigned to inpatient consultation. This exposure provides them with an in depth understanding of NHSN definitions and how to identify and communicate HAI reduction opportunities. Fellows also meet with IPs and the data manager to understand the reporting software advantages and limitations and HAI validation methods. They regularly attend CLASBI, CAUTI, and CDI HAI reduction task-force meetings, where they learn HAI reduction strategies in the context of other patient safety events and help to identify high-risk patient populations. In addition to participation in the HAI task-force meetings, they attend the OSUWMC IPC, individual hospital infection prevention work groups and other system-wide quality meetings that are deemed relevant by the medical directors.

### Cluster detection, investigation, and resolution

Fellows are required to complete the *Primer on Healthcare Epidemiology, Infection Control and Antimicrobial Stewardship: Online ID Fellows Course* by the SHEA^4^ at the beginning of their rotation, which provides an excellent background for cluster and outbreak investigations. Fellows meet with the IPs individually to discuss the exposure notification process for significant pathogens including tuberculosis, varicella, scabies, and SARS-CoV-2.

Active participation in cluster detection, outbreak investigation, and subsequent follow-up is a strength of the EPI track. Both fellows who completed the EPI track participated in a significant cluster investigation. Our 2019–2020 fellow investigated a cluster of postpartum group A *Streptococcus* infections on the labor and delivery unit^2^ and our 2020–2021 fellow investigated a cluster of COVID-19 on an inpatient hospital unit involving multiple modes of transmission.^3^ Through these investigations, the fellows learned the role of case–control and cohort investigation and how to conduct an exposure investigation. Following both of these investigations, each fellow has had the opportunity to present their respective investigation, findings, and interventions to various institutional stakeholders and leaders and at national meetings.

### Pathogen transmission and transmission interruption

The competencies outlined by Martin and Snyder^1^ are addressed during both the EPI rotation and track. Hand hygiene, modes of pathogen transmission, and rationale for isolation are taught starting in the first year of fellowship and are reinforced during the SHEA training course. These aspects are further reinforced during the rotation and track activities when meeting and performing rounds with the IPs and when reviewing the OSUWMC isolation and hand hygiene policy. General HAI reduction strategies are reviewed in the SHEA compendium, and institutional strategies are discussed during individual meetings with the IPs, during HAI task-force meetings and the IPC. In addition, fellows join the IPs during unit rounding where hand hygiene, personal protective equipment, and isolation practices are observed in real time. Fellows on the EPI track review OSUWMC cleaning and low-level disinfection policy, the high-level disinfection policy and round with the IPs and HLD analysts in areas performing high- and low-level disinfection.

### Additional competencies

Many of the additional competencies outlined by Martin and Snyder^1^ are included as part of the program activities (Table 1) including environment of care, occupational health and emergency preparedness. The EPI department maintains a close working relationship with the clinical microbiology and antimicrobial stewardship departments, and fellows participate in meetings and projects involving these departments in addition to dedicated training in these departments as part of their fellowship training.

### Academics and scholarly activities

All of the fellows are required to present their quality research project at both the enterprise-wide IPC and the ID conference series. Depending on the topic, they may present at other sessions, including grand rounds, the Central Ohio APIC chapter meetings, and the SHEA and IDSA conferences.

Once the EPI track is completed, the fellow receives a graduate medical education (GME) certificate recognizing the successful completion of the subspecialty training. This certification is valuable for future employment opportunities and provides recognition that can be understood by administrators.

## Discussion

Here, we describe our institution’s EPI ID fellow education using both a 1-month rotation and 1-year training track. In review of the model outlined by Martin and Snyder,^1^ our program includes many of these core competencies with a focus on experiential learning. Our program’s strengths include strong training in surveillance and reporting, close collaboration with the IPs, dedicated mentoring by the medical director faculty, and real-time cluster and outbreak investigations. All fellows participate in a quality improvement project, but additional training on the principles of quality improvement and project design would be beneficial as well as additional experience in program implementation. A future consideration would be utilizing a postgraduation survey to identify additional opportunities for program improvement. Providing EPI training during ID fellowship is critical, especially given the heightened awareness of infection prevention due to the COVID-19 pandemic. The 2 fellows who have completed the track are working in clinical epidemiology roles at major academic centers. Our program has been successful in providing this training to all fellows and more comprehensive training for fellows interested in pursuing a career in healthcare epidemiology.
